# Pesquisa nacional para a avaliação da abordagem à lesão do LCA entre cirurgiões esportivos brasileiros

**DOI:** 10.1055/s-0045-1812464

**Published:** 2025-11-18

**Authors:** Eduardo Frois Temponi, Lúcio Honório de Carvalho Júnior, Matheus Braga Jacques Gonçalves, Pedro Martins da Costa Drummond, Vitor Rodrigues de Miranda, Waldir de Souza Fernandes Júnior

**Affiliations:** 1 Hospital Madre Teresa, Belo Horizonte, MG, Brasil

**Keywords:** inquéritos e questionários, joelho, ligamento cruzado anterior, reconstrução do ligamento cruzado anterior, anterior cruciate ligament, anterior cruciate ligament reconstruction, knee, surveys and questionnaires

## Abstract

**Objetivo:**

Descrever o perfil dos cirurgiões de joelho brasileiros e suas preferências no diagnóstico, tratamento e reabilitação da lesão do ligamento cruzado anterior (LCA), e fazer uma comparação com dados da literatura atual.

**Métodos:**

Realizou-se um levantamento eletrônico entre cirurgiões membros da Sociedade Brasileira de Artroscopia e Traumatologia Esportiva (SBRATE). Foram incluídos 746 ortopedistas com endereço cadastrado. Foi enviada mensagem com o convite para participação, os objetivos da pesquisa, e o *link* para acesso ao questionário, que continha 36 perguntas sobre perfil profissional, diagnóstico, técnica cirúrgica e reabilitação da lesão do LCA.

**Resultados:**

O número de participantes foi de 170 (22,7% dos 746 ortopedistas convidados). Todos os participantes realizavam reconstrução do LCA, sendo que 72% tinham mais de 10 anos de experiência, e 43,5% realizavam mais de 50 reconstruções anuais. O trauma esportivo foi o mecanismo de lesão mais comum (95%). Instabilidade (95,3%) e retorno ao esporte (82,2%) foram os principais critérios para indicação cirúrgica. A maioria (53,5%) indicava cirurgia entre a primeira e a quarta semanas após a lesão. Enxerto dos flexores (88,2%) e técnica transportal *in-out* (39,2%) foram as opções mais frequentes. No pós-operatório, carga imediata (51,8%) e arco de movimento completo precoce (61,3%) foram as abordagens predominantes.

**Conclusão:**

O estudo delineou o perfil e as condutas dos cirurgiões de joelho da SBRATE na reconstrução do LCA. Os achados indicam que as práticas no Brasil seguem as tendências globais descritas na literatura recente.

## Abstract (en)

**Objective:**

To describe the profile of Brazilian knee surgeons and their preferences in the diagnosis, treatment, and rehabilitation of anterior cruciate ligament (ACL) injuries, and to compare the findings with data from the current literature.

**Methods:**

We conducted an electronic survey among surgeons who were members of the Brazilian Society of Arthroscopy and Sports Traumatology (Sociedade Brasileira de Artroscopia e Traumatologia Esportiva, SBRATE, in Portuguese). The study included 746 orthopedic surgeons with registered email addresses. We sent a message with an invitation to participate, the research objectives, and a link to access the questionnaire, which contained 36 questions about the professional profile of the surgeons and their preferences in the diagnosis, surgical technique, and rehabilitation of ACL injuries.

**Results:**

The number of participants was 170 (22.7% of the 746 orthopedic surgeons invited). All participants performed ACL reconstruction, with 72% having more than 10 years of experience and 43.5% performing more than 50 reconstructions per year. Sports trauma was the most common injury mechanism (95%). Instability (95.3%) and return to sport (82.2%) were the main criteria for surgical indication. Most participants (53.5%) indicated surgery 1 to 4 weeks after injury. Flexor grafting (88.2%) and the in-out transportal technique (39.2%) were the most frequent options. The predominant postoperative approaches were immediate weight-bearing (51.8%) and early full range of motion (61.3%).

**Conclusion:**

The study outlined the profile and practices of SBRATE knee surgeons in ACL reconstruction. Our findings demonstrate that Brazilian practices follow global trends described in the recent literature.

## Introdução

A reconstrução do ligamento cruzado anterior (LCA) evoluiu significativamente nas últimas décadas. A técnica consagrada, com uso do enxerto patelar autólogo fixado por parafusos de interferência, perdeu hegemonia em levantamentos recentes. 1 2 3 Descrições anatômicas mais precisas, seguidas de estudos clínicos e biomecânicos, modificaram as tendências atuais em vários aspectos do tratamento. 4 5 O conceito de reconstrução anatômica redefiniu as metas de restauração ligamentar, ao demonstrar melhores resultados quando comparados aos das reconstruções isométricas. 6 7 Tópicos como perfuração do túnel femoral, tipo do dispositivo de fixação, preservação dos remanescentes, manutenção da inserção tibial dos tendões flexores e reconstrução extra-articular combinada são amplamente debatidos. Embora diferentes métodos apresentem particularidades técnicas, quando executados adequadamente, mostram eficácia comparável 8 9 10 e impacto positivo na qualidade de vida. 8 11

Os protocolos para diagnóstico, tratamento e prognóstico da lesão do LCA são constantemente atualizados. Estudos de nível I de evidência estabelecem padrões de qualidade e guiam decisões clínicas, embora os relatos dos pacientes frequentemente divirjam das avaliações médicas, o que dificulta a análise objetiva dos resultados. 11 12 Apesar da ampla produção científica, fatores externos influenciam a conduta dos cirurgiões na prática diária. 1 No Brasil, diferenças econômicas e sociais impactam as escolhas terapêuticas, dada a disparidade entre os sistemas público e privado. 13 O aumento da incidência das lesões do LCA, ligado a novos padrões de atividade, reforça a importância de compreender as práticas atuais no Brasil. 12 Nos Estados Unidos, estima-se entre 200 mil e 250 mil lesões anuais, com custo aproximado de 13 mil dólares por cirurgia. 14

Os questionários de pesquisa são valiosos para coleta de dados e apresentação das práticas, atitudes e tendências. São usados por pesquisadores para análise quantitativa dos dados que são essenciais na epidemiologia clínica e serviços de saúde. 15 16 Uma revisão sistemática de Ekhtiari et al. 17 incluiu 20 questionários sobre práticas na cirurgia de reconstrução do LCA em várias regiões do mundo. No Brasil, há poucos dados descritivos sobre a lesão do LCA. Ambra et al., 1 em estudo realizado durante um evento nacional, relataram que a escolha dos cirurgiões depende do tempo de experiência e da viabilidade de recursos. Este estudo visa descrever o perfil epidemiológico da reconstrução do LCA no Brasil e compará-lo com levantamentos globais que abordam tópicos controversos na literatura.

## Materiais e Métodos

O estudo foi aprovado pelo comitê de ética da nossa instituição sob o número CAAE: 78506324.6.0000.5127 e pela Comissão da Sociedade Brasileira de Artroscopia e Traumatologia do Esporte (SBRATE).

Foi realizado um levantamento (questionário) online com ortopedistas especializados em reconstrução do LCA, membros da SBRATE. Um questionário estruturado com questões relacionadas ao perfil epidemiológico das reconstruções do LCA no Brasil foi elaborado na plataforma Google Forms (Alphabet Inc.). Antes da sua aplicação, o instrumento passou por um processo de validação de conteúdo, sendo revisado por um grupo de cirurgiões especialistas em joelho da instituição de origem, com o objetivo de garantir clareza, relevância e adequação das perguntas ao contexto clínico nacional. O convite para participação, termo de consentimento, objetivo do trabalho e *link* para acesso ao questionário foram enviados por e-mail a 746 membros cadastrados. O primeiro envio, em 5 de janeiro de 2023, foi aberto por 249 membros, e 79 deles clicaram no *link* . Em um segundo envio, após 14 dias, 231 membros abriram a mensagem, e 41 clicaram no *link* . O período de resposta permaneceu aberto até 5 de agosto de 2023 para maximizar a taxa de participação. As respostas foram anônimas, e o sistema impediu duplicações. O estudo foi constituído de um questionário detalhado ( **Apêndice 1** ) sobre o perfil dos cirurgiões e suas preferências. No total, foram elaboradas 36 questões com 2 padrões de resposta possíveis. Em 26 delas, os participantes escolhiam somente uma resposta, e, nas outras 10, podia-se selecionar mais de uma opção. As primeiras perguntas levantaram características demográficas dos participantes, assim como dados relativos à experiência profissional, seguidas de questões relacionadas aos parâmetros diagnósticos, abordagem e planejamento. As demais eram relativas a aspectos da técnica cirúrgica e reabilitação.

### Análise Estatística

Estatística descritiva foi calculada para cada parâmetro analisado. As variáveis contínuas foram apresentadas como médias, e as categóricas, como distribuição de frequências. Os resultados foram comparados com os de levantamentos recentes sobre tendências globais no tratamento da lesão do LCA.

## Resultados

### Perfil dos Cirurgiões

**Fig. 1:**
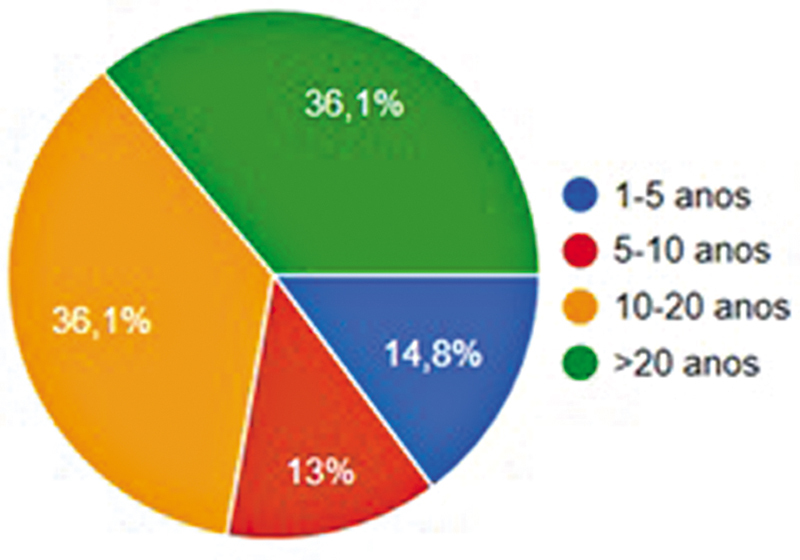
Tempo de experiência com a reconstrução do ligamento cruzado anterior (LCA).

**Fig. 2:**
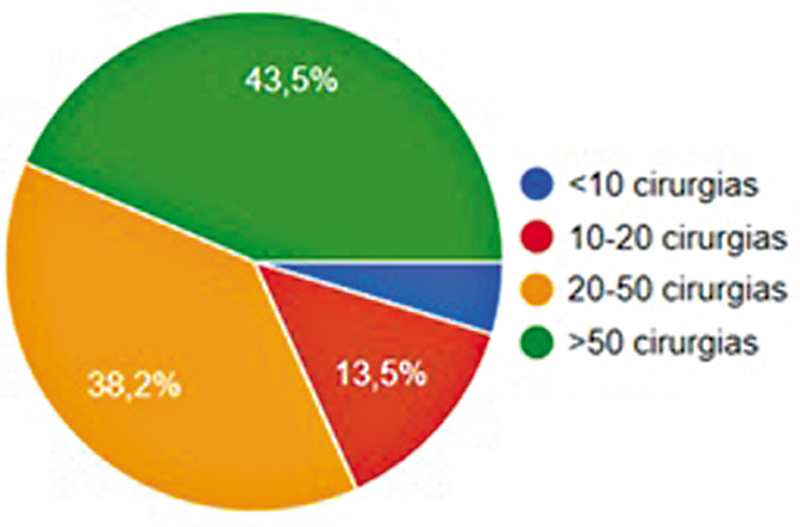
Número de reconstruções do LCA por ano.

Dos 746 ortopedistas afiliados à SBRATE com e-mail cadastrado, 170 (22,7%) responderam, sendo apenas 1,8% do sexo feminino. Todos os participantes confirmaram que realizavam a cirurgia de reconstrução do LCA. A média de respostas por questão foi de 167,7. A faixa etária predominante foi a de 40 a 50 anos (36,1%), e 14,8% tinham mais de 60 anos. A maioria dos participantes (80%) exercia a prática ortopédica havia mais de 10 anos, e 72% tinham igual tempo de experiência com reconstrução do LCA ( Fig. 1 ). Além disso, 43,5% realizavam mais de 50 cirurgias anuais ( Fig. 2 ).

### Abordagem Diagnóstica

**Fig. 3:**
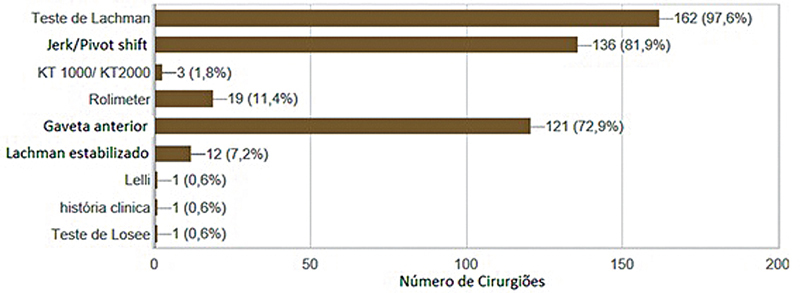
Parâmetros clínicos para diagnóstico.

O trauma esportivo foi a principal etiologia relatada (95,9%). As condutas mais comuns no trauma agudo foram o uso de gelo (84,7%) e medicação (60,4%). Exame clínico (93,5%) e ressonância magnética (86%) foram os métodos diagnósticos mais utilizados. Na ressonância magnética, o corte sagital foi considerado o mais adequado para o diagnóstico por 85% dos participantes, seguido do coronal (35,2%) e do coronal oblíquo (33,9%). Entre os testes clínicos, o de Lachman (97,6%), *jerk test* / *pivot shift* (81,9%) e gaveta anterior (72,9%) foram os preferidos ( Fig. 3 ).

### Técnica Cirúrgica e Reabilitação

**Fig. 4:**
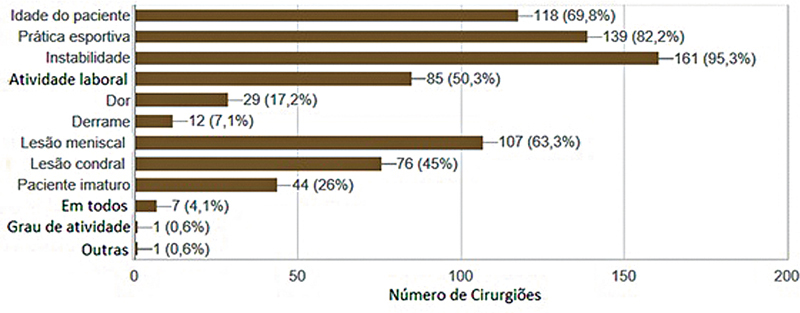
Fatores para indicação da reconstrução do LCA.

**Fig. 5:**
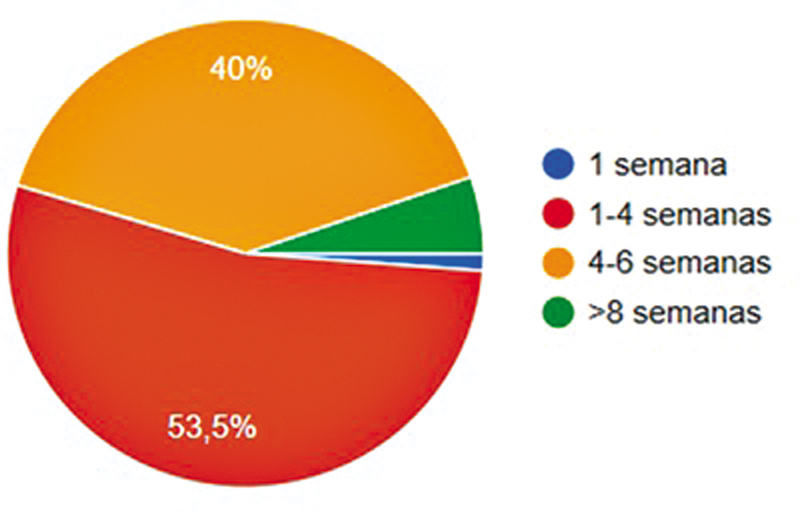
Tempo de preferência após a lesão para reconstrução do LCA.

A instabilidade no joelho relatada pelo paciente foi a principal indicação para o tratamento cirúrgico (95,3%), seguida da vontade de retornar à prática esportiva. Idade e lesão meniscal também foram parâmetros levados em consideração para a tomada de decisão cirúrgica ( Fig. 4 ). O momento preferido pela maior parte dos cirurgiões para realização da cirurgia foi entre 1 e 4 semanas ( Fig. 5 ). Raquianestesia (58,6%), uso de torniquete (98,2%) e tempo cirúrgico entre 30 e 60 minutos (68%) foram as práticas mais relatadas.

**Fig. 6:**
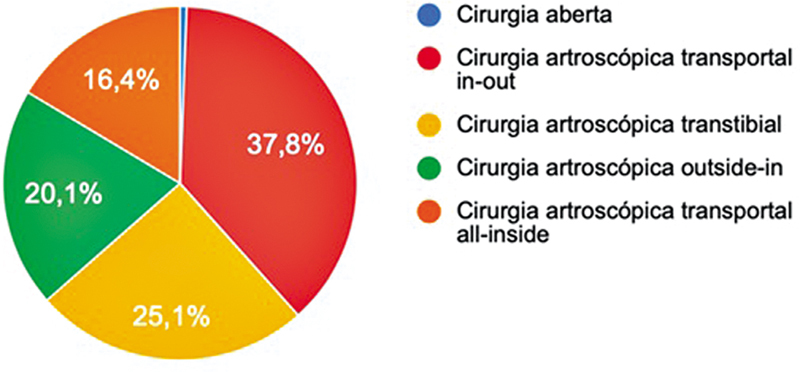
Técnica preferida para reconstrução do LCA.

O enxerto autólogo dos tendões flexores foi escolhido por 88,2% dos cirurgiões, e 72,6% realizavam pré-tensionamento. Já em relação à técnica de perfuração do túnel femoral, a via transportal *in-out* foi a escolhida por 37,8% dos cirurgiões, e a transtibial, por 25,1% ( Fig. 6 ). O uso do portal acessório medial foi relatado por 26,7%.

Diante da presença dos remanescentes ligamentares, 41% dos cirurgiões preservavam integralmente o coto tecidual, ao passo que somente 13,3% optavam por removê-lo completamente. A notchplastia era indicada por 41,7% nos casos de estreitamento do intercôndilo, e por 29,4% quando havia pinçamento do enxerto durante o arco de movimento. A reconstrução do ligamento anterolateral (LAL) foi citada por 11,6% dos cirurgiões, ao passo que 51,8% não realizavam o procedimento, e 34,1% somente o indicavam em situações específicas. O parafuso de interferência foi o implante mais utilizado para fixação (77,6%), com preferência pelo modelo absorvível. Dispositivos de fixação por suspensão com botão ajustável foram escolhidos por 22,4% dos participantes.

**Fig. 7:**
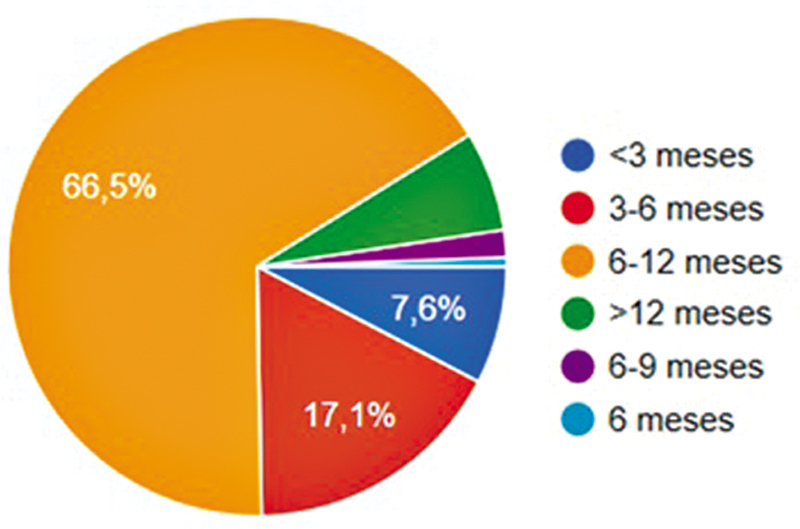
Tempo para liberação de atividade física após a reconstrução do LCA.

**Fig. 8:**
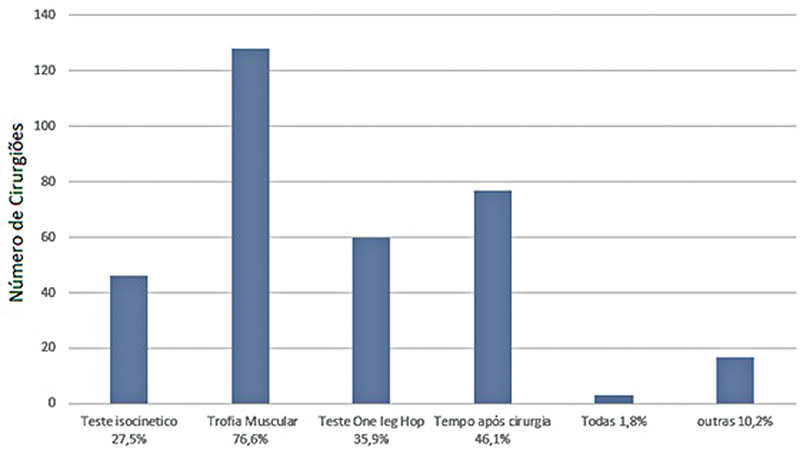
Parâmetros para retorno ao esporte.

A fisioterapia pós-operatória era rotina para 99,4% dos ortopedistas. A liberação da carga imediata foi relatada por 51,8% dos participantes, e 61,3% não impunham restrição ao arco de movimento. Apenas 10,2% recomendavam órteses no pós-operatório. A liberação para atividade física ocorria após 6 a 12 meses para 66,5% dos cirurgiões ( Fig. 7 ), e 59,3% relataram que esse era o tempo esperado pelos pacientes. Os principais critérios para retorno foram trofismo muscular (76,6%), tempo de pós-operatório (46,1%) e teste *single-leg hop* (35,9%) ( Fig. 8 ).

**Fig. 9:**
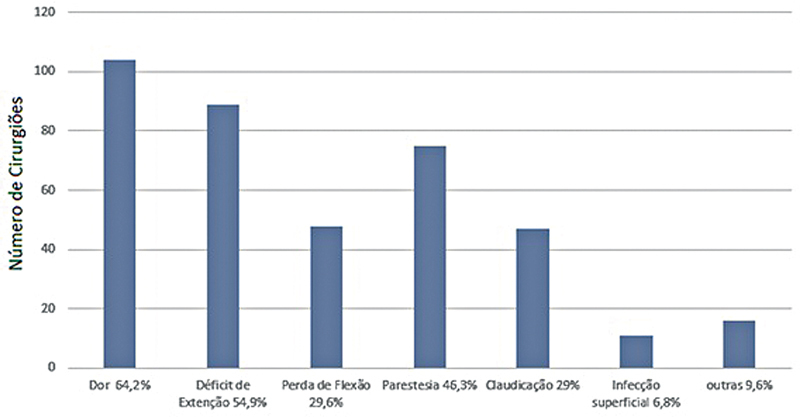
Complicações de curto prazo.

**Fig. 10:**
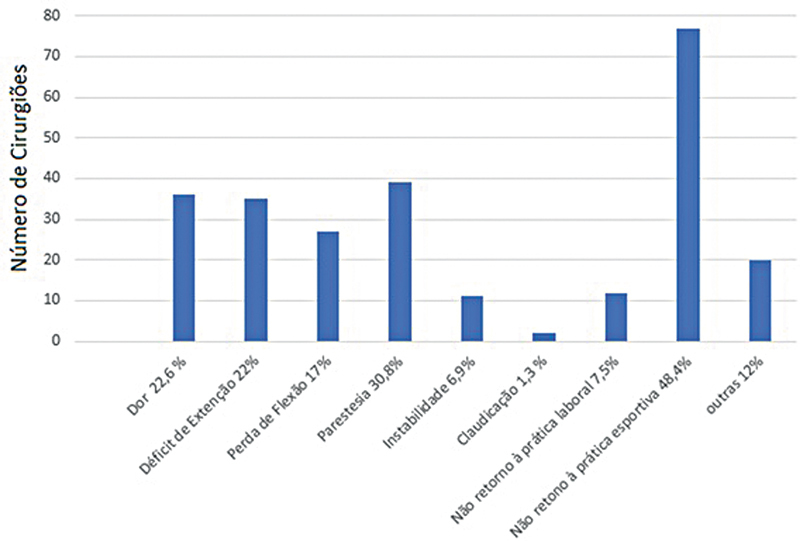
Complicações de longo prazo.

As complicações de curto prazo mais relatadas foram dor (64,2%), déficit de extensão (54,9%), parestesia (46,3%), perda de flexão (29,6%), claudicação (29%) e infecção superficial (6,8%) ( Fig. 9 ). Após 6 meses, a principal complicação foi o não retorno à atividade física (48,4%), seguido de parestesia (30,8%) ( Fig. 10 ). Houve redução na frequência de dor, déficit de extensão, perda de flexão e claudicação nesse período.

## Discussão

O objetivo deste levantamento foi investigar o perfil e as preferências dos cirurgiões especialistas na reconstrução do LCA que atuam no Brasil. Pesquisas semelhantes entre membros de sociedades foram realizadas em várias partes do mundo, 1 2 12 18 19 mas este é o primeiro levantamento brasileiro a mapear as tendências de 170 cirurgiões peritos na reconstrução do LCA. Entre os participantes, 72% realizavam reconstruções havia mais de 10 anos e 43% realizavam mais de 50 procedimentos anuais. Erickson et al., 20 em um levantamento feito nos Estados Unidos com cirurgiões experientes dos times da liga de futebol americano (National Football League, NFL, em inglês) em 2014, encontraram uma média de 82 reconstruções por ano e tempo de experiência médio de 16,8 anos, ao passo que Shafizadeh et al. 3 apontaram uma média de 35 por ano, e diferenciaram cirurgiões de alto (acima de 50 reconstruções por ano) e de baixo volume. Os resultados mostraram diferenças significativas entre eles em relação à filiação em sociedades, à duração do procedimento, ao uso de exames de estabilidade e à técnica de perfuração preferencial. Os parâmetros mais citados por eles para indicação da reconstrução foram a instabilidade subjetiva e o teste de Lachman. Poucos relataram o uso de instrumentos de medida da estabilidade: 17% mencionaram o Rolimeter (Aircast Europa), e 10%, o KT1000 (MEDmetric Corporation). A maioria preferiu o período entre 6 semanas e 3 meses para a reconstrução.

Neste estudo, a instabilidade (95%) e a prática esportiva (82%) foram os principais fatores de indicação cirúrgica, com 93% dos especialistas preferindo operar entre a primeira e a sexta semanas após a lesão. No levantamento de Farber et al. 21 com médicos das equipes de Liga de Futebol dos Estados Unidos (Major League Soccer, MLS, em inglês), 48% dos cirurgiões faziam a reconstrução nas 2 primeiras semanas, e 33%, entre a segunda e a quarta semanas. McRae et al. 18 buscaram estabelecer tendências por meio do nível de concordância entre as respostas dos cirurgiões canadenses. Falseio (instabilidade do joelho) para atividades diárias e físicas, alta demanda e lesão meniscal reparável atingiram valores superiores a 80% de concordância como critérios para indicação do tratamento. Área doadora ipsilateral à lesão, técnica com incisão única e pré-tensionamento também atingiram esses níveis.

A opção pelo enxerto autólogo dos flexores foi alta (88,2%) neste estudo, embora a literatura 22 ainda debata qual é o melhor tipo de enxerto. Em uma revisão sistemática, Reinhardt et al. 23 compararam reconstruções com enxertos dos tendões patelar e flexores, e observaram maior taxa de falha nos flexores (15,8% contra 7,2%, respectivamente), ao passo que o patelar apresentou mais dor anterior. Um levantamento brasileiro anterior 1 encontrou taxa ainda maior, com 93% optando pelos flexores. Chechik et al. 24 relataram variações regionais: 72% dos europeus e 42% dos norte-americanos preferiam os flexores. Farber et al. 21 apontaram que 68% dos cirurgiões usaram patelar autólogo em jogadores da MLS. Erickson et al. 20 relataram preferência de 86% dos cirurgiões pelo uso do tendão patelar nos atletas da NFL, e de 50% em atletas recreacionais com idades acima de 25 anos. Cerciello et al., 25 em um novo questionário aplicado a jovens cirurgiões europeus, apontaram que o uso do tendão do quadríceps está ganhando popularidade entre praticantes de esportes de contato, ao passo que o enxerto de tendões do semitendinoso e do grácil são preferidos em pacientes de baixa demanda.

Os resultados deste estudo confirmam a consolidação da perfuração independente do túnel femoral no Brasil, o que se alinha à tendência global. Embora a literatura ainda não demonstre superioridade clínica, 26 somente 16,3% dos cirurgiões ainda utilizam a técnica transtibial, percentual inferior ao do levantamento nacional anterior, 1 no qual 26,5% a preferiam, o que torna esse dado ainda mais relevante. Nenhum cirurgião com mais de 15 anos de experiência optou pela técnica transportal anteromedial. Em estudos internacionais, 24 68% dos especialistas preferiam a via transportal anteromedial e 31%, a transtibial, ao passo que Erickson et al. 20 relataram 67% de preferência pela perfuração pelo portal medial acessório, e 25%, pela transtibial. Farber et al. 21 identificaram que 50% dos cirurgiões ainda utilizam a técnica transtibial. Outra mudança relevante identificada foi a maior preservação do remanescente ligamentar. Somente 13,3% dos participantes ainda realizavam a remoção completa do coto. Em 2011, McRae et al. 18 encontraram correlação entre a preservação e os cirurgiões com maior volume de reconstruções no Canadá; no entanto, entre todos os participantes, 45% ainda sacrificavam integralmente o remanescente.

A reconstrução do LCA associada ao procedimento extra-articular lateral pode melhorar o controle da estabilidade rotacional do joelho em relação à reconstrução isolada. No entanto, sua indicação permanece controversa devido às incertezas sobre o real ganho em estabilidade e o perfil ideal dos pacientes beneficiados. Atualmente, a reconstrução do LAL é preferida em casos de *pivot shift* de alto grau e revisões cirúrgicas. 27 Tramer et al. 28 relataram que 59,6% dos cirurgiões não realizavam a reconstrução do LAL junto ao LCA, resultado semelhante ao deste estudo, em que 51,8% dos entrevistados não adotavam a técnica.

Os resultados sobre reabilitação mostram que cirurgiões brasileiros adotam protocolos acelerados, com liberação precoce de carga e arco de movimento completo, tendência semelhante à de 77% dos participantes no estudo de Campbell. 29 Resultado semelhante foi encontrado por McRae et al.: 18 72% liberavam carga total e 75% arco de movimento total imediatamente após a cirurgia. Um questionário com 46 cirurgiões americanos aplicado por Glattke et al. 30 sugeriu que uma alta porcentagem de cirurgiões utilizava órteses pós-operatórias, por um período que pode variar de 3 a 6 semanas. Este, no entanto, ainda é um tema de considerável variabilidade entre os protocolos adotados por cirurgiões ortopédicos.

Levantamento realizado por Petersen e Zantop 19 elencou critérios para retorno ao esporte competitivo, sendo que 63,5% recomendavam retorno após 6 meses e 76,6% iniciavam exercícios específicos em 4 meses, principalmente com base no teste de Lachman negativo e arco de movimento total. Outros parâmetros incluíram testes proprioceptivos (43%), *single-leg hop test* (39%) e força muscular (40%). No entanto, 85,8% dos participantes do estudo não seguiam parâmetros específicos. Neste estudo, mais de 2/3 dos cirurgiões brasileiros recomendaram retorno entre 6 e 12 meses, o que se alinha ao estudo de Vascellari et al., 2 que também identificaram recomendação de retorno entre 6 e 8 meses em 73% dos casos. Com 4 semanas, 33% permitiam bicicleta estacionária, e somente 16% liberavam *jogging* após 8 semanas. Erickson et al. 20 observaram que 74,5% dos cirurgiões permitiam retorno ao esporte aos atletas da NFL com base no *single-leg hop test* , 55,4% sugeriam o tempo mínimo de 6 meses após a cirurgia, e 56% requeriam arco de movimento normal, ausência de dor, força normal e estabilidade. 20 Em revisão sistemática de Mayer et al., 31 destaca-se a importância dos testes funcionais para o retorno seguro ao esporte, embora não haja consenso sobre quais testes são os mais eficazes.

Estudo de Grassi et al. 32 avaliou as recomendações para retorno ao esporte após a reconstrução do LCA. O tempo de 6 meses para retorno foi citado por 87% dos participantes para esportes sem contato, e por 43% para esportes de contato. No nível competitivo, as taxas de retorno em 6 meses caíram para 48% para os esportes sem contato, e para 13% para os de contato. Não houve diferença significativa quanto ao tipo de enxerto ou experiência cirúrgica. Arco de movimento total, além dos testes de Lachman e *pivot shift* , foram os critérios mais utilizados, com 90% dos cirurgiões adotando pelo menos um parâmetro. Somente metade dos entrevistados utilizava avaliação instrumental da força muscular, e 30% aplicavam testes funcionais. O uso de órtese somente foi recomendado por 10% dos participantes, o que contrasta com a taxa de 49% relatada por Vascellari et al. 2 Farber et al. 21 observaram maior frequência de uso da órtese em reconstruções com enxerto dos flexores. Segundo Mayer et al., 31 a falta de padronização nos protocolos de reabilitação e nos testes de retorno ao esporte é um desafio contínuo, e mais pesquisas de alta qualidade são necessárias para estabelecer recomendações baseadas em evidências.

Este foi o primeiro questionário online aplicado a cirurgiões especialistas no tratamento de lesões do LCA no Brasil. A qualificação dos participantes conferiu confiabilidade aos dados. A principal limitação foi a baixa taxa de resposta (22,7%). Estudos semelhantes na Itália, como os de Grassi et al. 32 e Vascellari et al., 2 obtiveram taxas de 16% e 17%, respectivamente. Outros levantamentos internacionais contaram com menos de 50 participantes na amostra. 13 21 Erickson et al. 20 registraram 51% de participação entre 267 cirurgiões de referência da NFL. Aspectos metodológicos relacionados ao sucesso do levantamento foram citados na revisão sistemática de Ekhtiari et al., 17 que investigaram a qualidade das pesquisas sobre a reconstrução do LCA. Foram incluídos 53 estudos e encontrada taxa média de respostas de 73%; os autores 17 concluíram que a adesão varia conforme o público e o método de distribuição, sendo maior em pesquisas presenciais e direcionadas a pacientes.

## Conclusão

Este estudo descreveu o perfil e as preferências dos cirurgiões de joelho membros da SBRATE que realizam a reconstrução do LCA do joelho. A instabilidade do joelho foi a principal indicação de tratamento cirúrgico; além disso, o enxerto autólogo dos tendões flexores foi a principal escolha entre os participantes, e o trofismo muscular, o parâmetro mais citado para o retorno à atividade física. Os resultados mostram que os cirurgiões brasileiros parecem incorporar as tendências globais descritas nos levantamentos recentes.
